# The Physiology of Vision

**Published:** 1841-07

**Authors:** 


### PART SECOND.

### Bibliographical Notices.

---

Art. I.—*The Physiology of Vision.* By William Mackenzie, m.d., Surgeon Oculist in Scotland in Ordinary to her Majesty the Queen. —*London,* 1841. 8vo, pp. 292.

Since the publication of Dr. Porterfield's admirable Treatise on the Eye, eighty-two years ago, no work on the subject of any consequence has appeared in this country. In Müller's Physiology, translated by Baly, we have, indeed, an excellent summary of the physiology of vision; but a separate work was still a desideratum in our medical literature; at least, a work which, being the production of one who knows mathematics as well as anatomy, might be consulted by the student without the risk of his being seriously led astray by any heterodox statement in either of these departments of science. It is scarcely necessary for us to mention to our readers that the mere mathematician has committed sad blunders from not knowing the structure of the eye, and the anatomist from ignorance of optics. For example, a mathematician tells us that the centre of the spherical surface of the retina and the centre of the lesser spherical surface of the cornea are the same; but were we to draw an eye on this principle, we should have a very odd delineation. An anatomist, on the other hand, will perhaps state that the lens is the principal agent of refraction in the eye, and that consequently when it is lost, as after operation for cataract, the refractive powers of the eye are greatly weakened. Now the cornea, and not the lens, is the principal agent of refraction in the eye, and the power of the lens is insignificant in comparison. But we can assure the student that he will find no such inaccuracies in the volume before us: on the contrary the work will prove to him a very safe and intelligible guide in the study of one of the most interesting of subjects to the contemplative mind. Dr. Mackenzie gives as a motto a quotation from Sir John Herschel, with which, as it points out so admirably the beauty of the study of the organ of vision, we will conclude this notice.

"It is the boast of science to have been able to trace so far the refined contrivances of this most admirable organ; not its shame to find something still concealed from its scrutiny; for however anatomists may differ on points of structure, or physiologists dispute on modes of action, there is that in what we *do* understand of the formation of the eye so similar and yet so infinitely superior to a product of human ingenuity; such thought, such care, such refinement, such advantage taken of the properties of natural agents used as mere instruments for accomplishing a given end, as force upon us a conviction of deliberate choice and premeditated design, more strongly, perhaps, than any single contrivance to be found, whether in art or nature, and render its study an object of the deepest interest."

